# A Multi-Year Longitudinal Study of Water Quality Parameters in Four Salmon-Bearing and Recreational Streams on Mount Hood, Oregon

**DOI:** 10.1371/journal.pone.0070453

**Published:** 2013-08-05

**Authors:** Ronald Wasowski, David Alexander, Steven Kolmes, Nathan Banet, Amy Card, Morgan Haines, Elijah Kolmes, Kelly Northcutt, Derek Roemer, Sarah Webber

**Affiliations:** 1 Environmental Science Department, University of Portland, Portland, Oregon, United States of America; 2 Biology Department, University of Portland, Portland, Oregon, United States of America; 3 Department of Fisheries and Wildlife, Oregon State University, Corvallis, Oregon, United States of America; 4 Columbia River Management Program, Oregon Department of Fish and Wildlife, Salem, Oregon, United States of America; 5 Mathey College, Princeton University, Princeton, New Jersey, United States of America; 6 The Environmental Center, Bend, Oregon, United States of America; 7 St. Mary’s School, Medford, Oregon, United States of America; 8 Oregon Health and Science University, Portland, Oregon, United States of America; University Medical Center Utrecht, Netherlands

## Abstract

Four streams–Clear Fork, Lost Creek, Camp Creek and Still Creek–in northwestern Oregon’s Sandy River Basin were monitored for temperature, dissolved oxygen levels, and fecal bacterial concentrations in a multi-year analysis examining stream health for recreational users and anchor habitat for Pacific Salmon. Temperatures were recorded using *micro* –*T* temperature loggers at 15 locations, during 22 July - 5 September 2006, 2 July - 4 September 2007, 20 June - 7 September 2008, 23 June - 9 September 2009, and 2 July –9 September 2010. The Seven-Day Average Maximum water temperature (7-DAM) of 13°C was used as a reference value for the biological limit governing suitable salmonid spawning and egg incubation conditions. The maximum 7-DAM temperatures occurred on different dates and all streams neared or exceeded the 13°C standard at least once each summer. Dissolved oxygen levels were measured at weekly or longer intervals in 2006, 2007, 2008, and 2009. Dissolved oxygen levels fell below the 9.0 ppm standard for Clear Fork on almost half the sampling dates in 2006, 2007, and 2009. Concentrations of the bacterial genus *Enterococcus* were measured as an indicator of fecal contamination. Samples were collected at 15 sites along the four streams. Weekly samples were collected during a 9 week period from July - September 2007, an 11 week period from June - September 2008, and an 11 week period from June - September 2009. *Enterococcus* counts exceeded the federal recommended national criterion value of 61 colony forming units (CFU) per 100 mL every year in Camp Creek and occasionally elsewhere, with exceedances trending towards late summer.

## Introduction

This four-year study examined characteristics of four streams on the western slopes of Mount Hood, Oregon, that are both salmon-bearing and recreational. The presence of campgrounds, cabins, a wastewater treatment plant, and spawning salmonids listed as threatened under the Endangered Species Act provides a complex and interesting milieu for interactions. Our measurements were of water quality parameters that impact both salmonid health and the health of campers in the Mount Hood National Forest who use the streams for recreational purposes.

Endangered Species Act listings have increased attention and funding for restoring degraded watersheds for threatened Pacific salmon *Oncorhynchus* spp. and steelhead *Oncorhynchus mykiss* [1]. However, wild salmon stocks have continued to decline since the mid-1800s [2]. The Sandy River Basin Working Group [3] confirmed that habitat degradation is the most important stressor on salmonid habitat within the Sandy River basin. The Sandy River basin supports spawning habitat for wild populations of spring and fall Chinook (*Oncorhynchus tshawytscha*), Coho (*Oncorhynchus kisutch*), and winter Steelhead (*Oncorhynchus mykiss*), all listed as threatened under the U.S. Endangered Species Act in the lower Columbia River [4]. The Oregon Department of Fish and Wildlife has implemented a Native Fish Conservation Policy [5].

This study focused on examining salmonid habitat by measuring three known indicators of habitat quality: water temperature, dissolved oxygen, and bacterial concentration. Human activities (e.g. riparian deforestation, and waste treatment plants) often alter water quality attributes. Salmonids coping with a stressor in one category such as thermal stress are less capable of adapting to additional stressors [6].

Brannon *et al.* [7] indicated that the poikilothermic nature of salmonids requires particular attention in determining how to maintain or restore appropriate thermal regimes. Temperature directly influences metabolic rate, growth, and additional physiological factors [8]. Poole *et al.* [9], [10] emphasized water temperature as a determinant in overall water quality of individual streams and entire watersheds. Streams such as those in the Sandy River basin, buffered by groundwater sources, transfer cool water in summer from aquifers to streams [11].

Negative consequences associated with elevated temperatures result in increased stress levels, inability to cope with additional stressors, disease, bioenergetic depletion, or lethality [12]. Thermal refugia, such as holes scoured out under logs during high flows, can shelter salmonids from thermally stressful conditions [12], [13]. The EPA [14] has provided upper optimal temperature threshold criteria for specific salmonid life cycles using a 7-day average maximum (7-DAM) of the daily temperature value. Excessive water temperatures pose a risk for salmonid spawning, rearing, migration, and smoltification depending on thermal stressor magnitude, duration, and frequency [12], [15]. High temperatures have been known to increase the virulence of pathogenic diseases in fish, increase metabolic rate, and decrease the water’s dissolved oxygen concentration [16]. Kocan *et al.* [17] found that disease rate increased with a progressive rise in temperature, producing negative consequences for migration stamina and evasion of predators during early life stages.

As another focus of this study, the presence of the bacterial genus *Enterococcus* is an indicator of fecal contamination which threatens human health and can contain many toxic materials that can bioaccumulate in salmonids. Enterococci are part of the normal intestinal microbiota of humans and many animals, and include species that are significant pathogens. Their presence in freshwater habitats indicates fecal contamination from a human or animal source [18]. They can be monitored relatively easily because cultivation on selective media at 41°C favors the growth of enterococci over that of other bacteria [19]. From human data, there is a direct correlation between the concentration of enterococci in recreational waters and swimming-associated gastroenteritis [20]. The EPA recommended recreational water quality standards for human health for *Enterococcus* in recreational freshwater were employed in this study [21].

While enterococcal diseases are not common in fish, salmonids are susceptible to infection by enterococci and other closely related bacteria under certain circumstances [22]. The presence of bacterial contamination impacts the stress load in stream systems and compromises resistance to disease [6]. One typical salmonid infection stems from the fungus *Saprolegnia parasitica* especially affecting spawning adults and juveniles under multiple stresses [23].

Dissolved oxygen concentrations are inversely related to water temperature, but they can increase due to aeration that occurs when water spalshes over rocks. For water in which salmonids spawn and incubate, the State of Oregon requires a minimum dissolved oxygen concentration in the water column of 9.0 ppm [24]. This is similar to the lower limits on dissolved oxygen set by the State of Washington, the Province of British Columbia, and most Northwestern Indian Tribes [25]. Lower dissolved oxygen levels can significantly impair salmonid reproduction and survival [26].

Monitoring the variables described above over the course of a multi-year study allowed us to document the variability in space and time of characteristics important to stream health for both fish and for people using the streams recreationally. We selected these variables over other environmental factors that impact streams because they had accompanying Federal standards and relative ease of quantification. Other variables, like shading and precipitation, lack Federal standards and are more difficult to quantify. For shading, its constantly changing nature at any location both across the course of a day and also across the summer pose a great challenge. Precipitation has very localized patterns on at different sites on Mount Hood that make quantification difficult (see for example Rhododendron and Government Camp precipitation data sets, accessible online through the National Climate Data Center).

## Materials and Methods

### Ethics Statement

We thank the personnel of the U.S. Forest Service's Mount Hood National Forest for permission to conduct this research.

### Site Description

**Figure 1:**
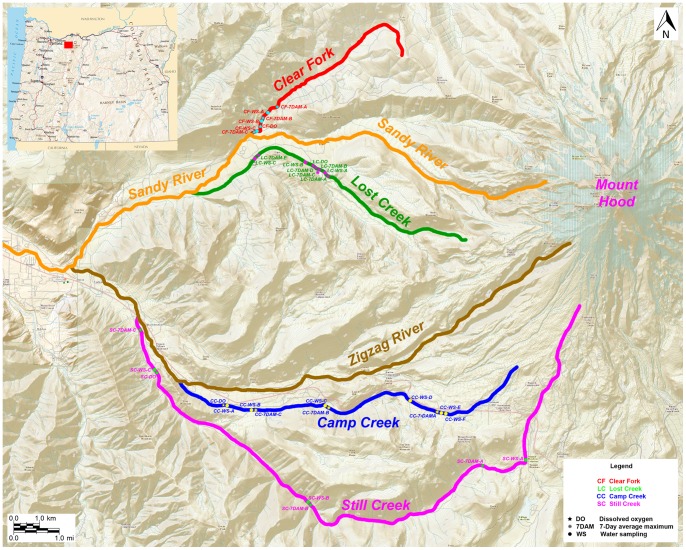
Map showing the location of the sampling sites in Oregon (insert) and along the four streams on Mount Hood. Stars indicate the location of dissolved oxygen sampling sites, asterixes indicate the location of temperature sampling sites, and circles indicate the sites for bacterial water sampling. Clear Fork and Lost Creek flow directly into the Sandy River, and Camp Creek and Still Creek flow into the Zigzag River, which is a tributary of the Sandy River.

This research was conducted during five consecutive summers from 2006–2010 (22 July – 5 September 2006, 2 July – 4 September 2007, 20 June – 7 September 2008, 23 June – 9 September 2009, and 2 July –9 September 2010) in Mount Hood National Forest. The National Forest Service manages an area encompassing 1,067,043 acres of temperate coniferous rainforest in northwestern Oregon. Our study sites were four streams within the Sandy River basin on the lower forested western slopes of Mount Hood, see Figure 1.

The streams in this study, Clear Fork, Lost Creek, Camp Creek, and Still Creek, are tributaries to the Sandy River, now a free-flowing tributary to the Columbia River. Streams varied in length from 1 km for Clear Fork to 5.3 km for Still Creek, the longest stream. Streams are fed either entirely or predominantly by groundwater sources and flow through forests characterized by Douglas-fir (*Pseudotsuga menziesii*), western hemlock (*Tsuga heterophylla*), and Pacific silver fir (*Abies amabilis*). Our sampling sites ranged in elevation from 1128.1 m to 482.5 m, see Table 1.

**Table 1:** Elevations and coordinates of all the sampling sites.

Feature	Latitude (°)	Longitude (°)	Altitude (m)
CF-7DAM-A	45.39776	–121.85796	683.4
CF-7DAM-B	45.39537	–121.85837	648.9
CF-7DAM-C	45.39172	–121.86169	641.3
CF-DO	45.39463	–121.85940	649.5
CF-WS-A	45.39644	–121.85770	651.1
CF-WS-B	45.39534	–121.85838	649.2
CF-WS-C	45.39184	–121.86086	644.3
LC-7DAM-A	45.37795	–121.82825	767.2
LC-7DAM-B	45.37870	–121.83244	722.4
LC-7DAM-C	45.37859	–121.83258	722.7
LC-7DAM-D	45.37881	–121.83278	722.1
LC-7DAM-E	45.38338	–121.86133	633.4
LC-DO	45.37899	–121.83310	721.5
LC-WS-A	45.37816	–121.82881	757.4
LC-WS-B	45.37954	–121.83389	720.9
LC-WS-C	45.38303	–121.86202	632.5
CC-7DAM-A	45.30156	–121.77817	1097.6
CC-7DAM-B	45.30412	–121.83291	743.7
CC-7DAM-C	45.30394	–121.86327	658.7
CC-DO	45.30345	–121.86949	636.7
CC-WS-A	45.30345	–121.86949	636.7
CC-WS-B	45.30386	–121.86359	657.5
CC-WS-C	45.30413	–121.83294	743.4
CC-WS-E	45.30648	–121.77173	1054.9
CC-WS-E	45.30141	–121.77768	1098.8
CC-WS-F	45.30131	–121.77731	1099.7
SC-7DAM-A	45.28491	–121.75734	1042.4
SC-7DAM-B	45.27307	–121.83722	731.8
SC-7DAM-C	45.33090	–121.91589	482.5
SC-DO	45.31534	–121.90752	525.8
SC-WS-A	45.28923	–121.73679	1128.1
SC-WS-B	45.27349	–121.83821	797.7
SC-WS-C	45.31534	–121.90752	525.8

Key:

Clear Fork (CF).

Lost Creek (LC).

Camp Creek (CC).

Still Creek (SC).

7-Day Average Maximum Temperature (7DAM).

Dissolved oxygen (DO).

Water sample (WS).

Spawning surveys conducted by the Oregon Department of Fish and Wildlife (ODFW) from 1996–1999 and 2002–2005 provide population estimates for the streams included in this study. The ODFW spawning survey records suggested productive salmonid spawning for Clear Fork and Still Creek in 1998 with redd densities of 28.3 redds/1.61 km and 27.9 redds/1.61 km respectively [27]. The ODFW surveys suggested relatively unproductive salmonid spawning in 1998 for Lost Creek and Camp Creek with records of 6.5 redds/1.61 km and 4.5 redds/1.61 km respectively [27]. All four streams maintained these productivity levels over time with the exception of Clear Fork having no redds in either 2002 or 2004, although redds were present all other years [27]. While Clear Fork was not surveyed in 2006 and 2007, counts in Lost Creek, Camp Creek and Still Creek in 2006 were consistent with productivity from prior surveys. In 2007, Lost Creek maintained its productivity with 4.5 redds/1.61 km while Camp Creek recorded 0 redds/1.61 km and Still Creek dropped to 8.5 redds/1.61 km [28].

The removal of the Marmot Dam in 2007, whose fish passage structures posed a challenge to migrating salmonids on the Sandy River, reestablished a free-flowing system. This increased the potential for utilization of historic spawning grounds including the four streams in this study, all of which are tributaries of the Sandy River upstream from the former Marmot Dam site. A comprehensive salmonid survey of Sandy River tributaries has yet to be completed following the removal of the Marmot Dam. Geomorphic and hydrologic data collected by the USGS after dam removal have been collected [29]. We thank the personnel of the U.S. Forest Service’s Mount Hood National Forest for permission to conduct this research. We thank them as well for access to normally inaccessible locations, and for occasional help with transportation.

### Temperature

The temperature sensors used for recording water temperatures were *micro-T* model DS1921G temperature loggers which employ *iButton* technology. These have +/−1°C accuracy, 0.5°C resolution, a range of –40°C to +85°C, and can record a maximum of 2,048 temperature measurements (a total of 85 days at one reading per hour). All *micro-T* loggers were calibrated before deployment using a laboratory-quality alcohol thermometer. In preparation for deployment, the *micro-T* temperature loggers were programmed to record measurements in degrees Celsius every hour on the hour. They were deployed in late July of 2006, early July of 2007, late June in 2008 and 2009, and early July of 2010. These dates were determined by water depth, and therefore site access, in the four streams. *Micro-T* loggers were retrieved in early September. Upon retrieval, the 7-DAM was calculated. By definition, a 7-DAM is the average of the maximum water temperature for seven consecutive days. The maximum 7-DAM for each site was graphed over the entire sampling period and compared to the 13°C 7-DAM upper optimal standard for spawning and egg incubation [12], [14].

In a typical deployment, two *micro-T* loggers were used at each site for redundancy. The *Parafilm® M*-wrapped *micro-T* loggers were encased in a perforated plastic golf ball sealed with a zip tie. This insured that the two loggers would remain together and be fully exposed to the current. The *micro-T* loggers were attached to cord-wrapped rocks coated with a layer of rubber on the bottom to prevent slipping of the cord. These rocks were then hidden in each stream in deep areas with swift water flow.

### Bacteriological Analysis

Weekly water samples were collected from 15 locations along the four streams. The samples were collected aseptically in sterile 120-mL polypropylene specimen containers. Randomized duplicate samples were obtained each week to confirm accurate counts. Each site was duplicated at least once during each season of the study. Field blanks of sterile water were also prepared at randomized sampling locations and lab blanks were prepared upon return to the laboratory to confirm that contamination did not occur during sample collection and analysis. All field samples and blanks were kept on ice in insulated coolers during transport to the laboratory. The samples were analyzed within 6 hours after collection of the first sample. Each water sample was vacuum filtered in aliquots of 100 mL through a 47-mm diameter Triton-free cellulose nitrate membrane filter with a pore size of 0.45 µm. To enumerate enterococci, the membrane filters were aseptically transferred to mE agar (Difco) and incubated for 48 hours at 41°C [19].

After incubation, the filters were transferred to esculin iron agar (EIA, Difco) and incubated for an additional 30 minutes at 41°C [19]. Bacterial colonies that produced red colonies on mE agar were identified as “suspected enterococcus” and those that also formed a black to reddish-brown precipitate when transferred to EIA were identified and counted as “confirmed enterococci.” The standard of a single sample maximal allowable density of 61 colony forming units (CFU)/100 mL for waters in which full body contact recreation takes place was used as the threshhold for excessive concentrations of enterococci. This standard is recommended for state and tribal waters of freshwater orgin to protect primary contact for safe recreation [21]. Young campers were frequently seen playing in the water of Lost Creek, Still Creek and Camp Creek.

Error was minimized with three independent counts of the colonies on each membrane filter, and high colony counts that were readable but nonetheless challenging by eye were confirmed by microscopic observation. Even with a microscope, colony counts above 300 colonies per plate could not be reliably made, and these values are represented in our data graphically simply at the 300 colony level and discussed in the text as exceeding the federally recommended water quality criterion for full body contact recreation of 61 CFU.

### Cross-sectional Stream Analysis Protocol for Dissolved Oxygen

For each stream analyzed, a cross-sectional template was employed. This template was made by stretching a rope across the stream and setting a series of eight equidistant points starting at the left bank and ending at the right bank. The template gave an objective and repeatable way to measure dissolved O_2_ since water levels and channel widths generally changed every week.

### Dissolved O_2_

The dissolved O_2_ meter used was a portable HACH HQ20. It has an O_2_ probe with an accuracy of +/−0.1 mg/L for concentrations between 0–8 mg/L and +/−0.2 mg/L for concentrations between 8–20 mg/L. The O_2_ probe was calibrated weekly prior to taking in-stream measurements. Measurements of O_2_ were made at the eight established equidistant points according to the cross-sectional stream analysis method. After the O_2_ levels were measured, a stream average was calculated for each week. All O_2_ measurements were made in the water column rather than making intragravel measurements which are highly correlated with water column measurements but more disruptive to the stream environment.

## Results

### Temperature

The 7-DAM values are graphed with the 13°C upper optimal threshold for salmonid spawning and egg incubation indicated by a red dashed line. Each 7-DAM value was associated with a particular stream measurement location. The sampling locations are encoded by the line color, indicated in the key by name and number (e.g. Lost Creek 7 DAM (A) is the most upstream site on Lost Creek) and indicated on the map provided as Figure 1. The streams are all graphed with early summer data to the left proceeding to later in the season as the axes go to the right. Temperature peaked at different times in different years, in a more unpredictable fashion than an assumption of streams growing warmer as the summer progressed would suggest.

**Figure 2:**
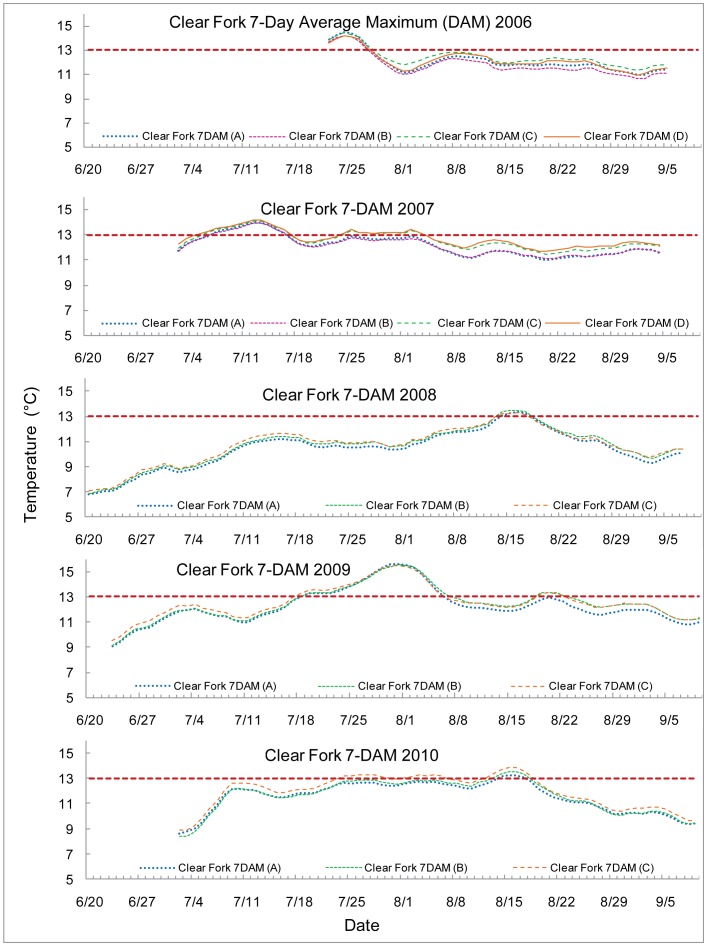
7-Day average maximum (7-DAM) temperatures of Clear Fork.

All temperature logger sites in Clear Fork were in close proximity to each other. Each maximum 7-DAM value exceeded the 13°C upper optimal threshold for salmonid spawning and egg incubation during one distinctive warming period each summer: the fourth week in July 2006, the second week in July 2007, the third week of August 2008, the fifth week of July 2009, and the third week of August 2010, see Figure 2. The summer of 2009 exceeded 15°C from July 29 - August 2, 2009. In 2007 and 2009, all sites were above the 13°C biological limit for 10 and 19 consecutive days respectively. Minimal temperature variability was observed between upstream and downstream sites.

**Figure 3:**
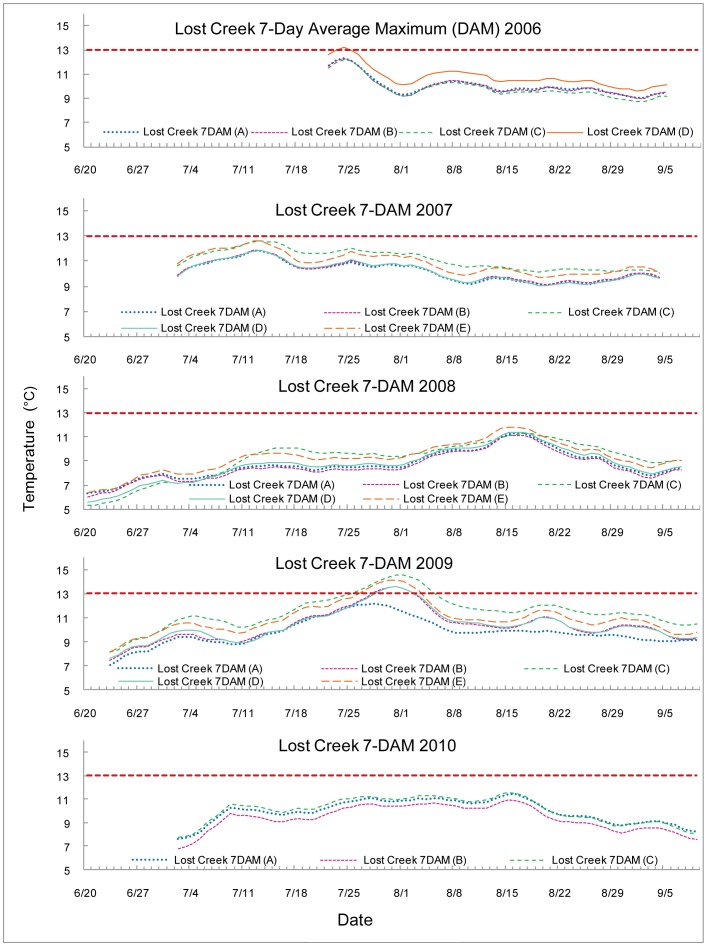
7-Day average maximum (7-DAM) temperatures of Lost Creek.

The Lost Creek 7-DAM maximum temperatures occurred at different times throughout the summer: the fourth week in July 2006, the second week in July 2007, the third week of August 2008, the fifth week of July 2009, and the third week of August 2010, see Figure 3. Site C was located on South Fork, a tributary to Lost Creek. The 7-DAM values exceeded the 13°C upper optimal threshold for the most downstream site, D, in 2006 and all but the most upstream site, A, in 2009.

**Figure 4:**
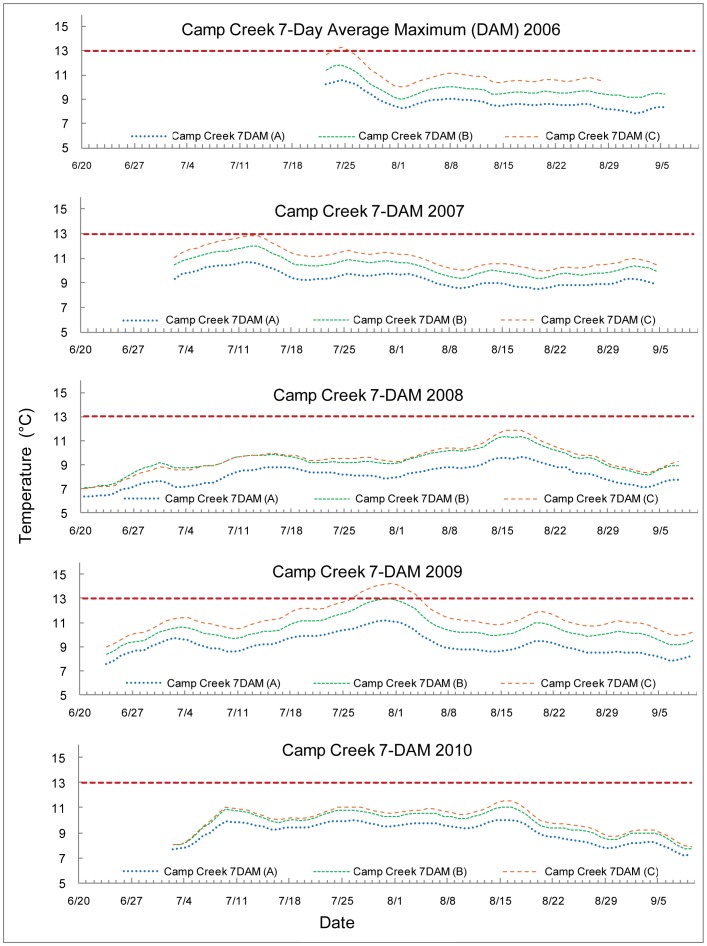
7-Day average maximum (7-DAM) temperatures of Camp Creek.

The Camp Creek 7-DAM maximum temperatures occurred at different times throughout the summer: the fourth week in July 2006, the second week in July 2007, the third week of August 2008, the fifth week of July 2009, and the third week of August 2010, see Figure 4. The most upstream site consistently recorded lower temperatures compared to the other two sites. Less difference was exhibited by the two sites downstream of site A in 2008 and 2010. The 7-DAM values exceeded the 13°C upper optimal threshold for the most downstream site, C, in 2006 and in 2009.

**Figure 5:**
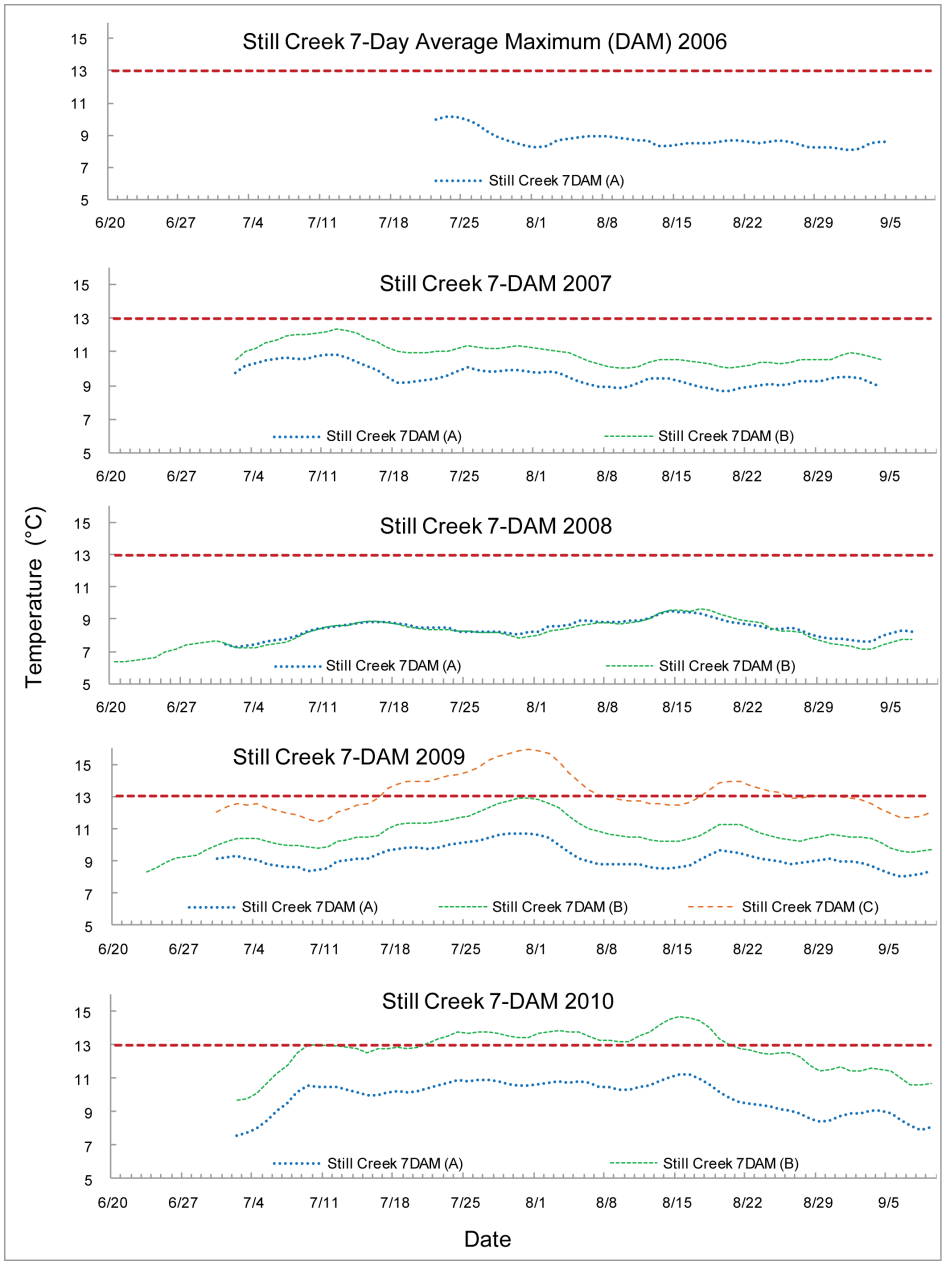
7-Day average maximum (7-DAM) temperatures of Still Creek.

Limited data were available for Still Creek. Values generally did not exceed the upper optimal threshold of 13°C in the first three years although sampling was limited due to unrecovered T-loggers. The 7-DAM maximum temperatures occurred at different times throughout the summer: the fourth week in July 2006, the second week in July 2007, the third week of August 2008, the fifth week of July 2009, and the third week of August 2010, see Figure 5. The maximum 7-DAM levels exceeded 15°C for the most downstream site, C, in 2009. The 7-DAM values for site C in 2009 and site B in 2010 exceeded the upper optimal threshold of 13°C for 22 consecutive days and 31 consecutive days respectively.

### Bacteria

The figures below display *Enterococcus* counts over the course of three sample years. In general Camp Creek was the most heavily contaminated of the four streams. However there was no stream in which the level of bacterial contamination was uniformly acceptable.

**Figure 6:**
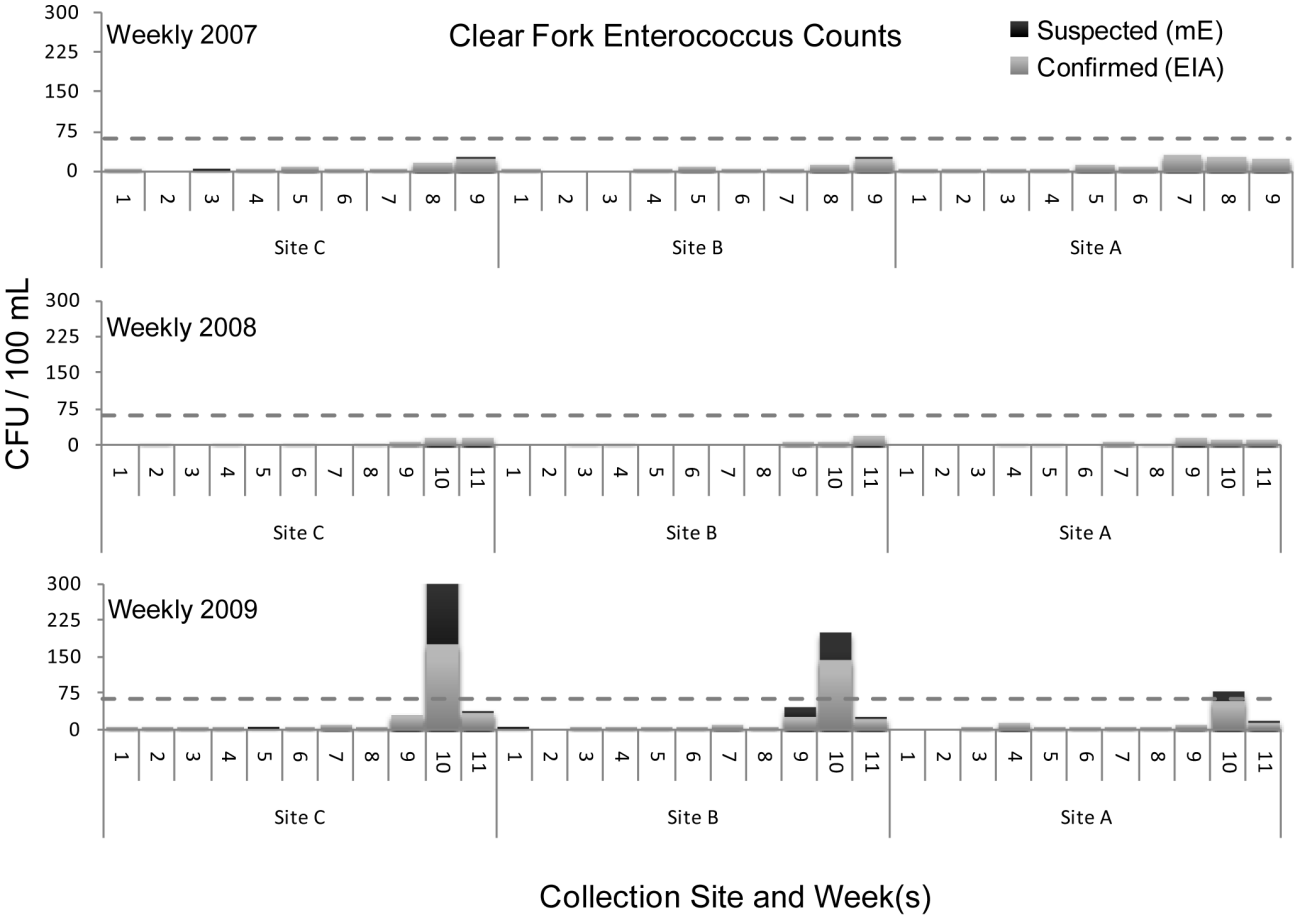
Clear Fork *Enterococcus* counts.

Clear Fork water samples all remained below the 61 CFU value for confirmed *Enterococcus* counts, the federally recommended water quality criterion for full body contact recreation, for all water samples in 2007 and 2008, but in 2009 exceeded that value in week 10 at sampling sites B and C, see Figure 6.

**Figure 7:**
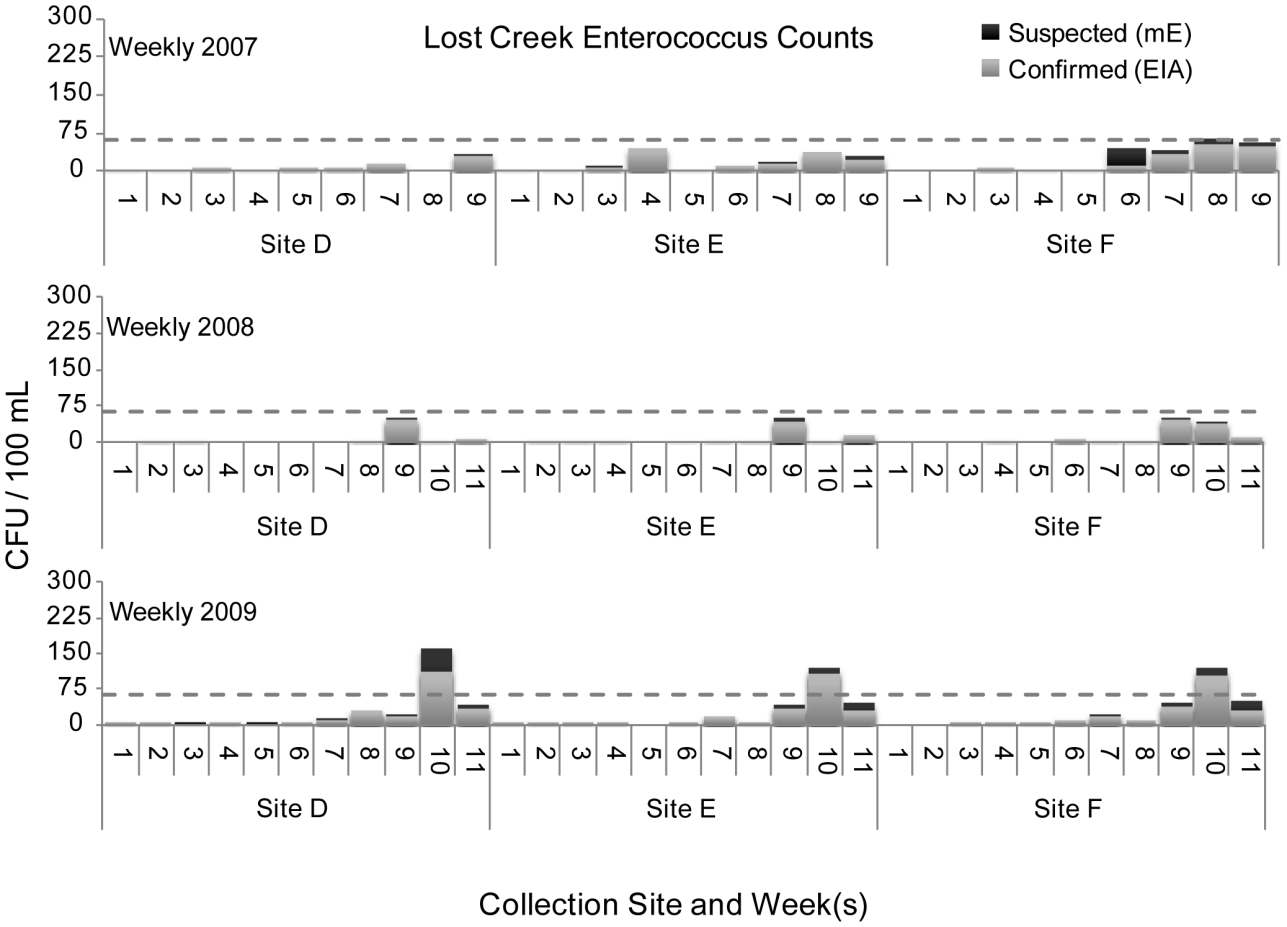
Lost Creek *Enterococcus* counts.

Lost Creek water samples all remained below the 61 CFU value for confirmed *Enterococcus* counts, the federally recommended water quality criterion for full body contact recreation, for all water samples in 2007 and 2008, but in 2009 exceeded that value in week 10 at sampling sites D, E, and F, see Figure 7.

**Figure 8:**
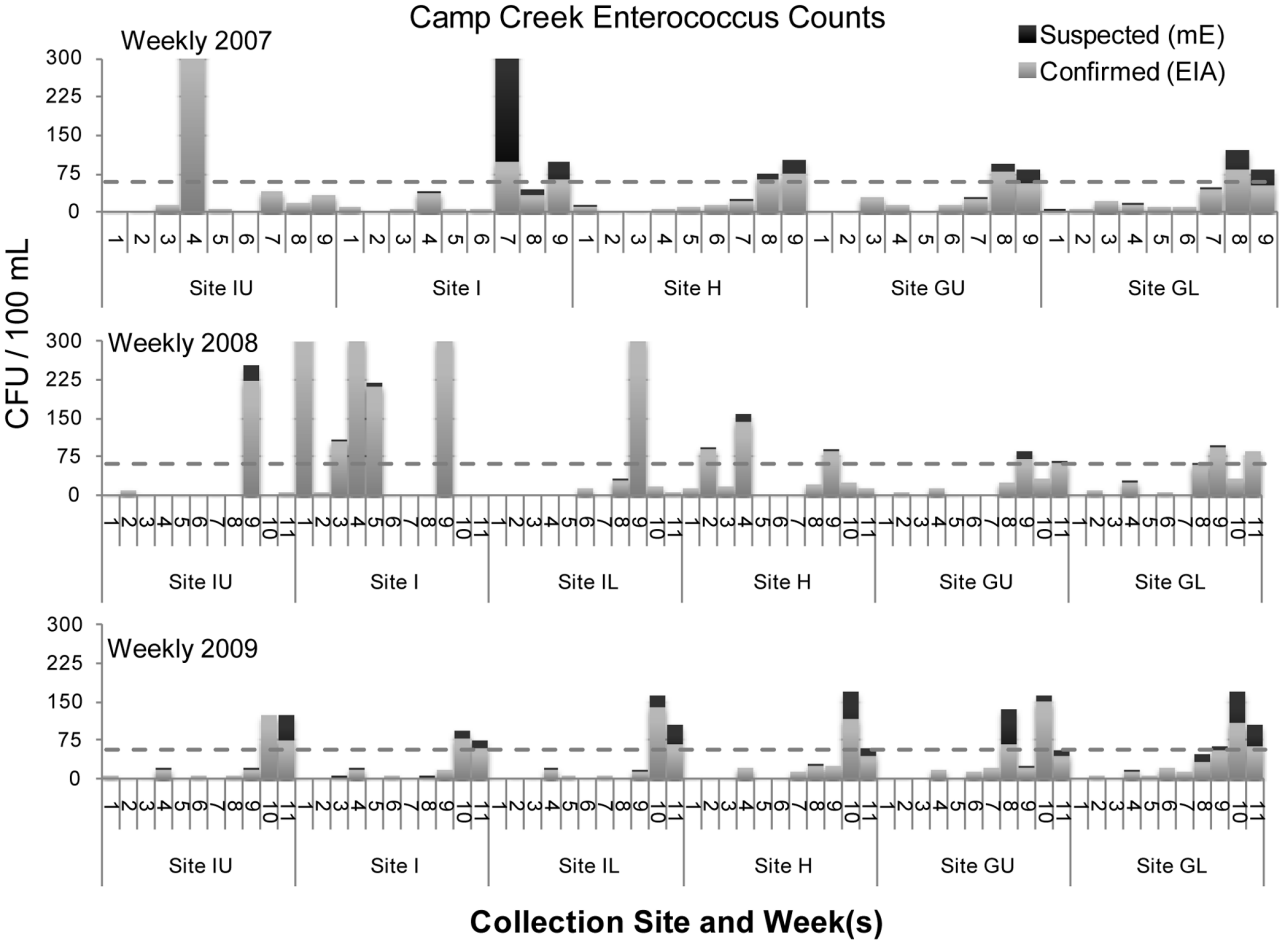
Camp Creek *Enterococcus* counts.

Camp Creek exceeded the 61 CFU value for confirmed *Enterococcus* counts, the federally recommended water quality criterion for full body contact recreation, in 2007 on week 4 at site IU, weeks 7 and 9 at site I, at weeks 8 and 9 at site H, at week 8 at site GU, and at week 8 at site GL. In 2008 counts exceeded 61 CFU at week 9 at site IU, at weeks 1, 3, 4, 5, and 9 at site I, at week 9 at site IL, at weeks 2, 4, and 9 at site H, at weeks 8 and 11 at site GU, and at weeks 9 and 11 at site GL. In 2009 counts exceeded 61 CFU at week 10 at sites IU, I, IL, H, GU, and GL. In 2009 counts exceeded 61 CFU at week 11 at sites IU, IL, and GL. The count equaled 61 CFU at site I at week 11, see Figure 8.

**Figure 9:**
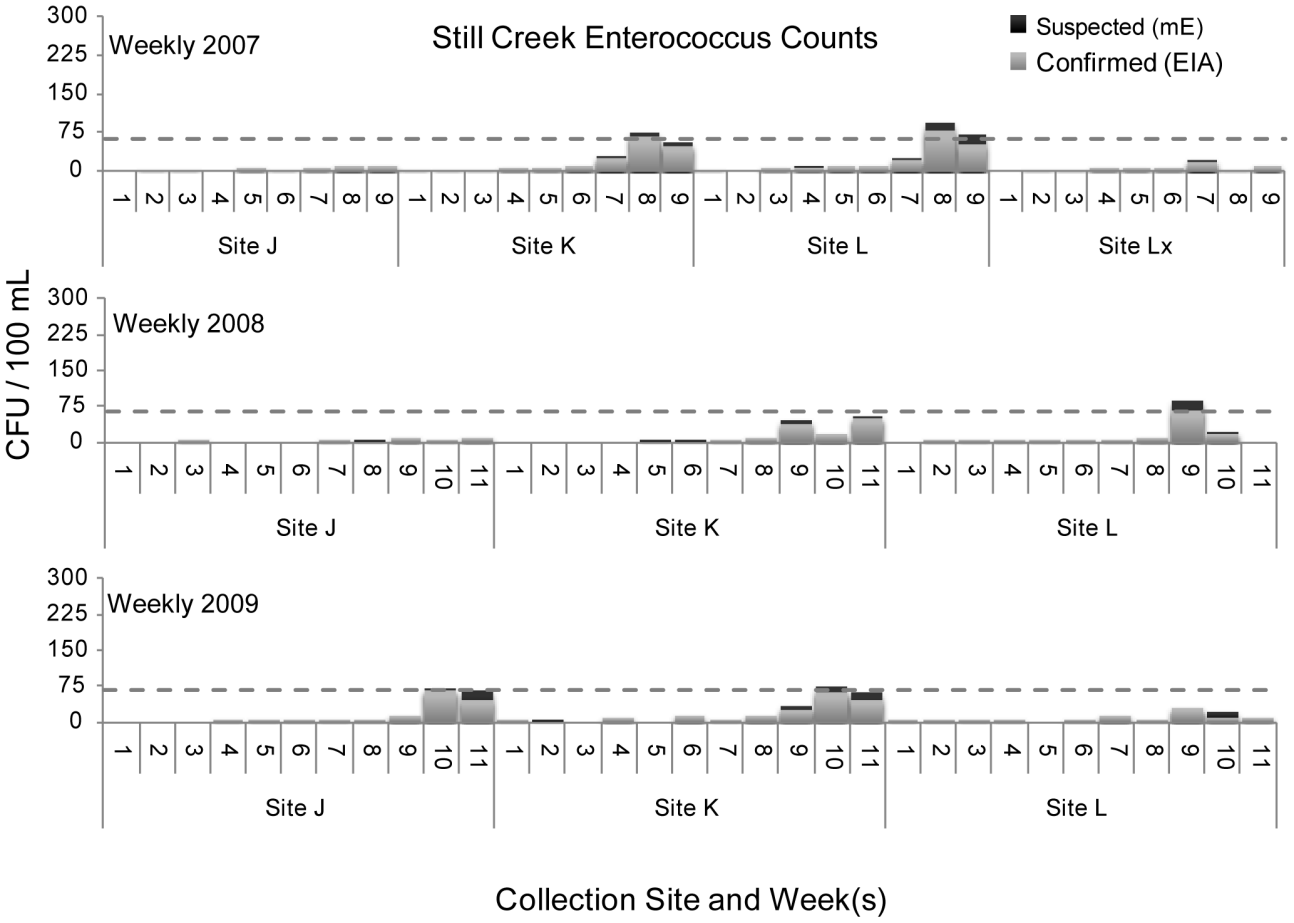
Still Creek *Enterococcus* counts.

Still Creek exceeded the 61 CFU value for confirmed *Enterococcus* counts, the federally recommended water quality criterion for full body contact recreation, in 2007 at week 8 at site K and at week 8 at site L. In 2008 counts exceeded 61 CFU at week 9 at site L. In 2009 counts exceeded 61 CFU at week 10 at site J, and equaled 61 CFU at week 10 at site K, see Figure 9.

### Dissolved Oxygen

Dissolved oxygen concentrations were generally within the acceptable range above 9.0 ppm for healthy salmonid habitat, with the exception of five instances in 2006, one instance in 2007 and six instances in 2009. The great majority of these low dissolved oxygen values were for Clear Fork. In 2006 Clear Fork registered 8.6 ppm on 6/21, 8.9 ppm on 7/7, 8.6 ppm on 7/21, and 8.4 ppm on 9/9. That year the South Fork of Lost Creek registered 8.9 ppm on 7/21 and 8.2 ppm on 9/9. No other measurements fell below 9.0 ppm in 2006. In 2007, despite a smaller number of dissolved oxygen measurements, Clear Fork registered 8.6 ppm on 7/6 and 8.9 ppm on 7/20. The South Fork of Lost Creek registered 8.7 ppm on 7/6. In 2008 no dissolved oxygen measurement fell below 9.0 ppm. In 2009 Clear Fork registered 8.4 ppm on 7/29, 8.4 ppm on 8/5, 8.6 ppm on 8/12, and 8.5 ppm on 8/19. The South Fork of Lost Creek measured 8.9 ppm on 7/29, and 8.8 ppm on 8/5. Clear Fork was the stream most commonly oxygen deficient, and low oxygen levels were more common later in the summer, see Tables 2, 3, 4, 5.

**Table 2:** Dissolved Oxygen 2006.

	6/7/06	6/20/06	6/21/06	6/23/06	6/28/06	7/5/06	7/7/06	7/21/06	9/9/06
CF	9.2	9.3	8.6	–	9.6	9.4	8.9	8.6	8.4
LCMAC	10.1	9.5	9.7	–	–	–	10.0	9.7	9.8
SF	9.2	–	9.0	–	–	–	9.3	8.9	8.2
LCMBC	9.6	9.5	9.4	–	9.7	9.8	9.7	9.4	9.6
CC	–	9.3	–	10.0	9.4	9.6	–	10.0	–
SC	–	9.3	–	9.7	9.4	9.7	–	9.7	–

Key:

Clear Fork (CF).

Lost Creek-mainstem above confluence of South Fork (LCMAC).

South Fork (SF).

Lost Creek-mainstem below confluence of South Fork (LCMBC).

Camp Creek (CC).

Still Creek (SC).

**Table 3:** Dissolved Oxygen 2007.

	6/22/07	6/28/07	7/06/07	7/20/07
CF	–	9.6	8.6	8.9
LCMAC	10.0	9.9	9.5	10.1
SF	9.3	9.4	8.7	9.0
LCMBC	9.9	9.7	9.5	9.9
CC	–	9.4	–	–
SC	–	9.4	–	–

Key:

Clear Fork (CF).

Lost Creek-mainstem above confluence of South Fork (LCMAC).

South Fork (SF).

Lost Creek-mainstem below confluence of South Fork (LCMBC).

Camp Creek (CC).

Still Creek (SC).

**Table 4:** Dissolved Oxygen 2008.

Dissolved Oxygen (mg/L) Cross Sectional Average 2008							
	6/27/08	7/11/08	7/18/08	7/25/08	8/1/08	8/8/08	8/15/08
CF	12.0	11.4	11.0	10.9	10.6	9.8	9.9
LCMAC	11.9	12.1	11.8	11.6	11.4	10.7	10.8
SF	11.6	10.3	10.7	10.7	10.5	9.9	9.9
LCMBC	11.6	11.2	11.6	11.4	11.3	10.6	10.5
CC	–	11.3	11.4	11.4	10.9	10.9	10.6
SC	–	10.7	10.8	10.9	10.7	10.7	10.2

Key:

Clear Fork (CF).

Lost Creek-mainstem above confluence of South Fork (LCMAC).

South Fork (SF).

Lost Creek-mainstem below confluence of South Fork (LCMBC).

Camp Creek (CC).

Still Creek (SC).

**Table 5:** Dissolved Oxygen 2009.

Dissolved Oxygen (mg/L) Cross Sectional Average 2009								
	7/1/09	7/8/09	7/15/09	7/22/09	7/29/09	8/5/09	8/12/09	8/19/09
CF	10.3	9.9	9.8	9.0	8.4	8.4	8.6	8.5
LCMAC	11.3	11.0	11.0	10.2	9.7	10.0	10.0	10.0
SF	10.5	10.3	10.2	9.4	8.9	8.8	9.3	9.0
LCMBC	11.0	11.2	10.8	10.0	9.5	9.8	9.8	9.7
CC	10.8	11.3	10.7	10.1	9.7	10.1	10.2	10.0
SC	10.2	10.7	10.0	9.6	9.3	9.6	9.8	9.7

Key:

Clear Fork (CF).

Lost Creek-mainstem above confluence of South Fork (LCMAC).

South Fork (SF).

Lost Creek-mainstem below confluence of South Fork (LCMBC).

Camp Creek (CC).

Still Creek (SC).

## Discussion

### Temperature Analysis

Thermal regimes of rivers are vitally important to the overall health of aquatic ecosystems, water quality, and the distribution of aquatic species [30]. For salmonids, thermal regimes are an especially critical environmental factor, with different life stages impacted at different temperature levels [8], [9], [10], [12], [16]. Spawning and incubation of eggs in the gravel is an especially sensitive life history stage. An upper optimal temperature of 13°C marks the biological limit governing suitable salmonid spawning and egg incubation conditions [12]. Peak summer temperatures that near or exceed 13°C would inhibit the abilities of salmonids to maintain a breeding population in a stream. Given the presence in the Sandy River basin of Coho salmon, fall-run Chinook salmon, spring-run Chinook salmon, Chum salmon, and Winter-run steelhead, spawning and subsequent incubation take place throughout much of the year [31]. In the Sandy River basin, winter–run steelhead may emerge from the gravel as late as the end of August, and fall-run Chinook may emerge as late as the beginning of July, so that incubating eggs are potentially present in streambed gravels throughout our study periods [31].

Clear Fork experienced high thermal stress for a historically productive anchor habitat [27], [28]. Each site on Clear Fork exceeded the 13°C biological limit at least once during the sampling period for the 7-DAM. This was not true for any other stream. Each site on Clear Fork recorded the highest 7-DAM values for each respective year compared to all other stream sites with the exception of Still Creek site C in 2009. Extended periods occurred in 2007 and 2009 in excess of the 13°C biological limit.

Lost Creek, an unproductive stream with low redd counts (27, 28) experienced considerable variability in temperatures from upstream to downstream sites. Lost Creek exceeded the 13°C threshold only twice, once in 2006 and once in 2009. All sites except the farthest upstream site in 2009 exceeded the standard. Lost Creek’s farthest downstream site experienced the greatest period in excess of the threshold.

Camp Creek, a relatively unproductive stream (27, 28), reported pronounced temperature variability between upstream and downstream sites. The most favorable temperatures, located at the farthest upstream site, would have been inaccessible to salmonids due to a large waterfall about 30 meters downstream of temperature logger site IL. This upstream site never approached the 13°C biological limit. Midstream and downstream temperature loggers approached or exceeded the 13°C biological limit.

Still Creek, in the summer of 2009, had the greatest temperature separation from upstream to downstream sites respectively compared to the other streams. Despite the success of salmonids in Still Creek [27], [28], the farthest downstream site in the summer of 2009 was of most concern out of all the temperatures recorded. This was the only site that exceeded the 13°C biological threshold on two separate occasions in one summer, the longest for 22 consecutive days. In 2010, the farthest downstream site exceeded the standard for 31 consecutive days.

### Dissolved Oxygen Analysis

The South Fork of Lost Creek experienced low dissolved oxygen levels at intervals, but this is a small tributary whose depth and flow rates would limit its usefulness to spawning salmon. The unexpected finding was that Clear Fork experienced frequent low dissolved oxygen levels for what was a historically productive anchor habitat (27, 28). Aside from the very shallow South Fork of Lost Creek, the other streams did not experience low dissolved oxygen levels.

### Bacteriological Analysis

Bacterial contamination varied between streams and within individual streams each summer (2007–2009). Concentrations of enterococci generally increased from early to late summer and the federal single sample *Enterococcus* recommended national criterion value of 61 CFU/100 mL for recreational waters was exceeded at various times each year.

Camp Creek was the most frequent tributary in this investigation to exceed the federal standard in the summer months with numerous spike events in excess of 61 CFU/100 mL above the single-sample limit (see Figure 8). Clear Fork’s bacterial spike events in excess of 61 CFU/100 mL above the single-sample limit occurred at two sites during the final two 5-week sampling periods in 2009. Lost Creek experienced a single spike event in excess of 61 CFU/100 mL, in week 10 of 2009. Bacteriological water quality remained within the acceptable range for Still Creek with the exception of one sampling period at sites K and L in the late summer of 2007 and at one site in the late summer of 2008.

Camp Creek’s high frequency of spike events in excess of 61 CFU/100 mL above the single-sample limit was due in part to a specific set of circumstances brought to light by our sampling. In 2008, a change in management at the Government Camp sewage treatment plant contributed to an improperly assembled discharge pipe. Direct discharge of raw sewage into Camp Creek ensued. At site I, the site about 15 meters downstream of the sewage discharge pipe, four of the sampling periods exceeded 300 CFU/100 mL and were deemed too high for accurate counting. Comparable levels of contamination were not detected at site IU, located about 15 meters upstream of upstream of the discharge pipe. The Government Camp sewage treatment plant was the only such facility in our sampling area, and contamination due to sewage runoff had previously been listed as a concern in the Sandy River basin [31].

Earlier studies have reported increased levels of fecal bacteria in campground streams associated with recreational activity [32], [33], [34]. Increases may have been due to stirred up sediment or waste from dogs and horses rather than to human waste [32], [33]. Along with the formal campgrounds equipped with pit toilets in the Mount Hood National Forest, like those on Camp Creek, Still Creek, and Lost Creek, informal or dispersed campsites are scattered along the streams and these lack sanitary facilities. Use of campsites lacking sanitary facilities has been shown to increase bacterial contamination of nearby streams, especially following weekends of heavy camping activity [34].

The Lost Creek Campground (45.38246, −121.83633) with 18 campsites and 2 vault toilets was above our sampling site E; also on Lost Creek the Riley Horse Camp (45.38139, −121.85944) with 14 campsites, 4 multiple horse corrals, ten hitching posts, and 2 vault toilets, was just above our sampling site F. Our sampling site GU was above the Camp Creek Campground (45.30409, −121.86708) with 28 campsites and 2 vault toilets, and our sampling site GL was below it. The Still Creek Campground (45.29583, −121.73556) with 27 campsites and 3 vault toilets was just above our sampling site J. Only Clear Fork lacked a formal campground that might serve as a source of bacterial contamination from people, dogs, or horses. Clear Fork, of the four streams, was the least frequent location in which bacterial samples exceeded the 61 CFU value for confirmed *Enterococcus* count.

### Conclusions

The Sandy River basin and its small tributary streams have been identified as very important to efforts to restore Chinook, Coho, and Steelhead [31]. This is considerably more important now than when the Sandy River Basin Characterization Report came out in 2005 [31], because the dams on the Sandy River have been removed and more habitat is easily accessible to returning spawners than had been for decades [29]. Elevated enterococci present in Camp Creek and Lost Creek is a transient but probably unavoidable problem [32], [33], [34]. Elevated water temperatures above the 7-DAM maximum temperatures were common but occurred at different times during different years. Clear Fork alone experienced frequent low dissolved oxygen levels. The environmental measurements reported in this article highlight the potential for restoration of wild salmonids in the Sandy River basin with even modest habitat recovery efforts. However, the ongoing Federal lawsuit brought by the Native Fish Society highlights the peril to wild fish posed by the unprecedented number of hatchery fish now able to reach spawning areas that were formerly inaccessible to them, due to the current regime of hatchery operations [35], [36], [37], [38], [39], [40], and the future of the four steams studied remains in doubt.
